# Genetic diversity among early provitamin A quality protein maize inbred lines and the performance of derived hybrids under contrasting nitrogen environments

**DOI:** 10.1186/s12863-020-00887-7

**Published:** 2020-07-18

**Authors:** Ebenezer Obeng-Bio, Baffour Badu-Apraku, Beatrice Elohor Ifie, Agyemang Danquah, Essie Takyiwaa Blay, Mustapha Abu Dadzie, Gilbert Tchala Noudifoulè, Abidemi Olutayo Talabi

**Affiliations:** 1 CSIR- Crops Research Institute, P. O. Box 3785, Fumesua, Kumasi, Ghana; 2 International Institute of Tropical Agriculture (IITA), PMB 5320, Oyo Rd, Ibadan, Nigeria; 3 West Africa Center for Crop Improvement (WACCI), University of Ghana, PBM 30 Legon, Accra, Ghana; 4 Cocoa Research Institute of Ghana, P.O. Box 8, New Tafo Akim, Ghana; 5 Institut Togolais de Recherche Agronomique (ITRA), B.P 1163/ Lomé Cacaveli, Lomé, Togo

**Keywords:** Genetic diversity, DArTseq markers, Provitamin A, Quality protein maize, Low-N tolerance, Inbred lines, Hybrids, *Zea mays *L.

## Abstract

**Background:**

Information on population structure and genetic diversity of germplasm in a breeding programme is useful because it enhances judicious utilisation of genetic resources to achieve breeding objectives. Seventy early maturing provitamin A (PVA) quality protein maize (QPM) inbreds developed by the IITA- maize improvement programme were genotyped using 8171 DArTseq markers. Furthermore, 96 hybrids derived from 24 selected inbreds plus four checks were evaluated under low-N and optimal environments in Nigeria during 2016 and 2017. Genotypic and phenotypic data of inbreds and hybrids respectively, were analysed to (i) assess the level of genetic dissimilarities and population structure of the inbreds, and (ii) investigate the grain yield performance of derived hybrids under low-N, optimal and across environments.

**Results:**

Genetic diversity among the seventy inbreds was high varying from 0.042 to 0.500 with an average of 0.357. Sixty-six inbred lines with probabilities ≥0.70 were assigned to a single group. The population structure analysis, the UPGMA phylogeny, and the principal Coordinate Analysis (PCoA) of the DArTseq markers revealed a clear separation of five groups and each followed pedigree records. Clustered inbreds displayed common characteristics including high PVA levels, and drought and low-N tolerance. The top performing hybrid, TZEIORQ 40 × TZEIORQ 26 out-yielded the best hybrid control, TZEIOR 127 × TZEIOR 57 by 8, 3, and 9% under low-N, optimal, and across environments, respectively. High repeatability estimates were detected for grain yield under each and across environments. Similarly, high breeding efficiency of 71, 70 and 72% were computed under low-N, optimal, and across environments, respectively.

**Conclusions:**

The UPGMA clustering, the structure analysis, and the PCoA consistently revealed five groups which largely followed pedigree information indicating the existence of genetically distinct groups in the inbred lines. High repeatability and breeding efficiency values estimated for grain yield of hybrids under low-N, optimal and across environments demonstrated that high productive hybrids could be developed using inbreds from the opposing clusters identified by the DArTseq markers. The 15 top performing hybrids identified, particularly TZEIORQ 40 × TZEIORQ 26 and TZEIORQ 29 × TZEIORQ 43 should be further evaluated for release and commercialization in SSA.

## Background

In plant breeding programmes, information on the genetic variation of germplasm is important because it facilitates judicious utilisation of resources to achieve breeding objectives. Genetic diversity in maize has always been exploited to select diverse parents to maximize heterosis in hybrids. Maize as an out-crossing species has a complex genome [1] with a high degree of genetic variability which is advantageous to the breeder in achieving high heterosis [2]. Semagn et al. [3] demonstrated that high genetic variation in a source population could enhance the development of useful inbred lines to aid the identification of best parental combinations for the development of superior hybrids. Studies have revealed tremendous variability among tropical maize germplasm. Zhang et al. [4] and Dao et al. [5] estimated gene diversity among tropical and temperate maize populations and found more diversity in the tropical than the temperate germplasm. The early maturing (90–95 days to physiological maturity) provitamin A (PVA)- quality protein maize (QPM) inbreds are novel tropical lines developed by the IITA-maize improvement programme with genes from diverse sources for PVA, quality protein, *Striga* resistance, and drought and low soil nitrogen tolerance. Assessment of the extent of genetic variability among the recently developed inbred lines would be useful in providing invaluable information to guide breeding strategies and facilitate progress in the development of hybrids and synthetics with combined drought and low-N tolerance, and high levels of PVA, tryptophan and lysine contents which are presently lacking in SSA. Maize production in SSA mostly occurs under low-N environments [6] by resource poor farmers who continuously crop maize with limited or no use of N fertilizer. During the past three decades, low-N has remained a great challenge to maize production and productivity in SSA resulting in about 10 to 50% loss of maize annually [7]. Therefore, the development and use of improved maize hybrids with tolerance to low-N conditions would contribute to superior grain yield potential in areas prone to low-N stress [8]. However, in the identification of low-N tolerant genotypes, the evaluations should be carried out under both low-N and favourable environments to accelerate gains from selection [9]. This approach enhances the identification of agronomically desirable genotypes that can give appreciable yield under low-N and maintain superior yield potential under favourable growing conditions. Moreover, heterosis could be maximized if the parental inbred lines involved in the hybrid combinations have varying genetic backgrounds [10]. It was therefore imperative to investigate the genetic backgrounds of the inbred lines in the present study using the Diversity Array Technology (DArT) which employs the Next Generation Sequencing (NGS) platform (DArTseq) [3, 11, 12] to provide high-density and cost-effective whole genome genotyping. Although, the DArTseq technique involves several steps in its delivery, it was the method of choice because it has the ability to provide genome profiles which are very useful for characterization of germplasm collections as well as reliable and precise phenotyping. The presumed genetic differences in the inbred lines coupled with natural genetic variability associated with tropical maize germplasm [4, 5] which could facilitate genetic improvement necessitated the assessment of the genetic diversity of the newly developed early maturing PVA-QPM inbred lines to ensure increased rate of genetic gain in derived hybrids. Thus, the present study was designed to (i) assess the genetic dissimilarities among the inbred lines using high-density DArTseq markers, (ii) examine the genetic structure of the inbred lines to maximize heterosis in hybrid combinations and (iii) investigate the performance of derived hybrids for grain yield and other agronomic traits under low-N, optimal and across environments.

## Results

### Summary statistics and phylogeny of inbred lines

**Fig. 1:**
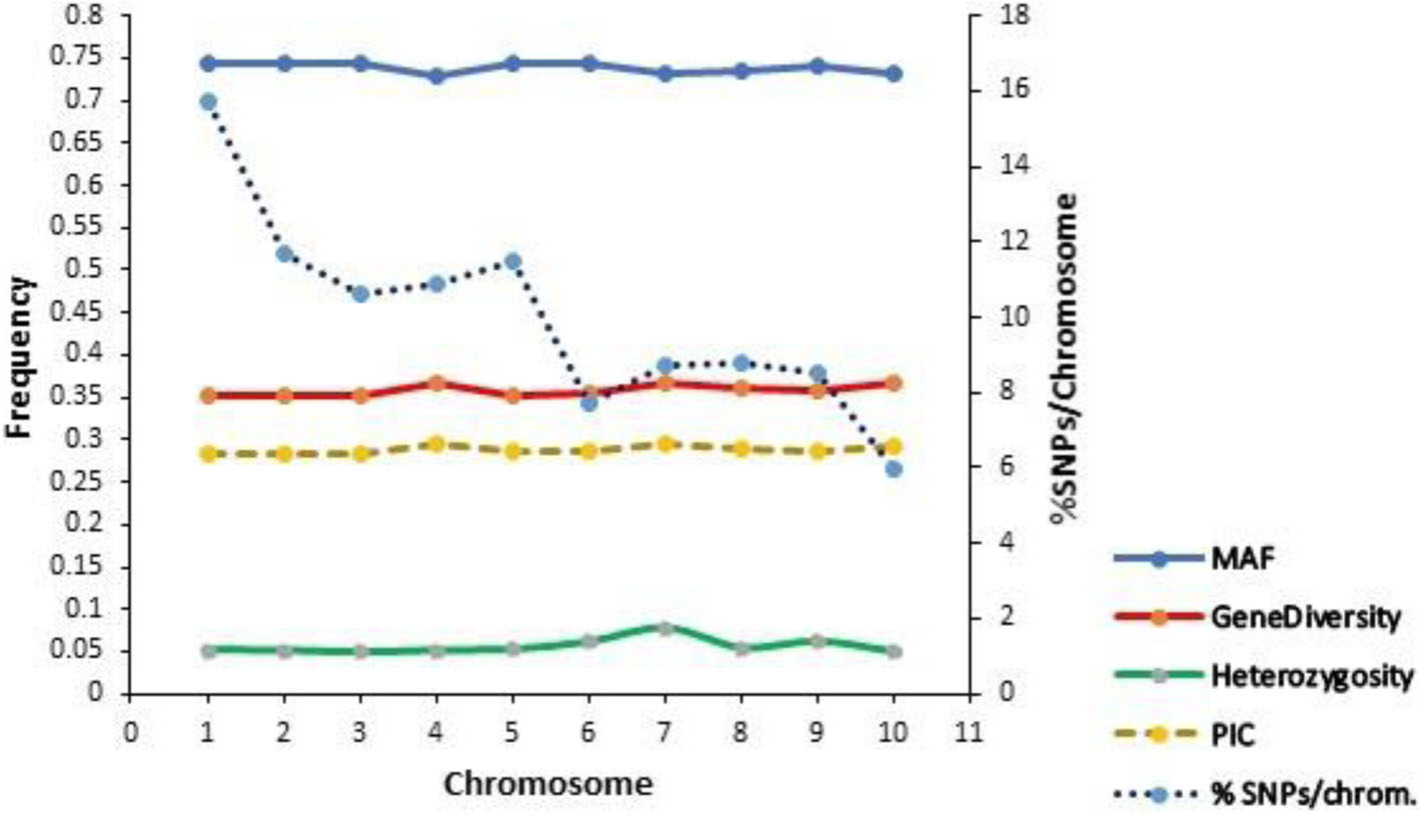
Summary description of the DArTseq markers used in the genetic diversity study of 70 early provitamin A-QPM inbred lines

In the subset of 8171 SNP-based DArTseq markers, changes in base pairs were A/C (892), A/G (2322), A/T (815), C/G (963), G/T (2278) and C/T (901). Among the polymorphic SNPs, the A/G and G/T transitions constituted the most informative which accounted for 28.4 and 27.9%, respectively. Gene diversity ranged from 0.042 to 0.500 with a mean of 0.357 (Fig. 1). A similar trend was observed for the PIC values which varied from 0.041 to 0.375 with a mean of 0.287. Heterozygous individuals identified per marker varied from 0.000 to 0.929 with a mean of 0.056. About 73% of the informative SNPs identified over 95% homozygous individuals. Major allele frequency ranged from 0.500 to 0.978 with a mean of 0.74. The genetic distance generated among the 70 inbred lines ranged from 0.018 to 0.455 with an average of 0.336.

**Fig. 2:**
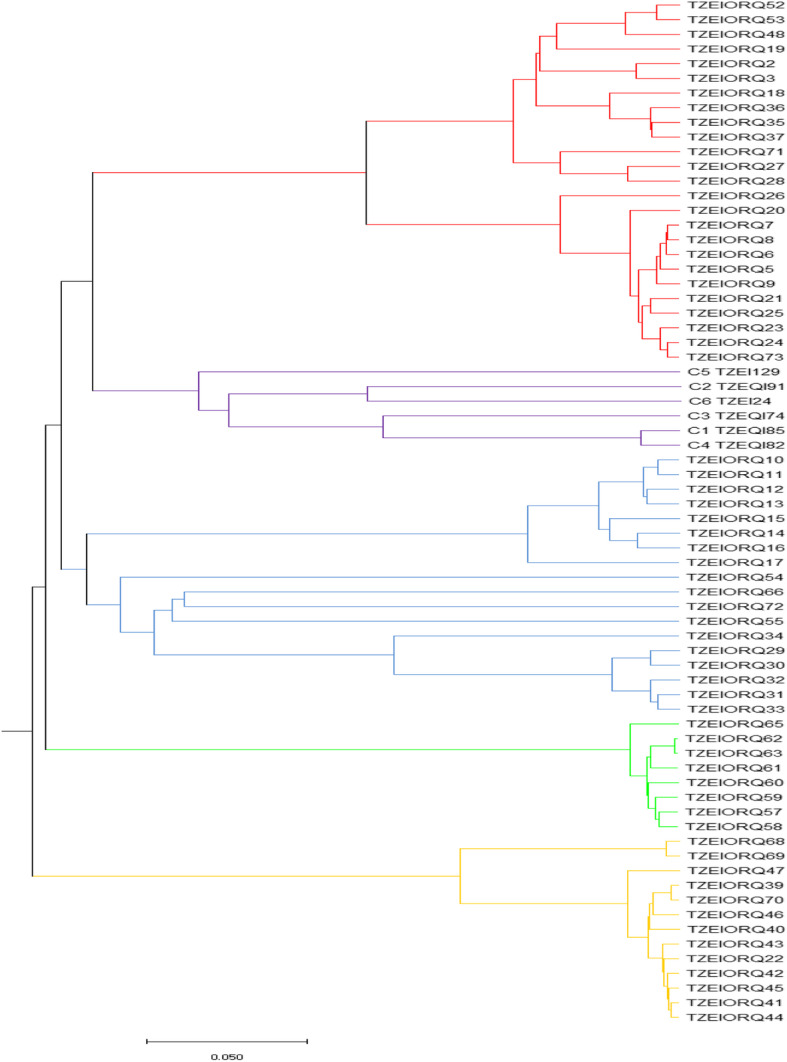
Phylogenetic tree of 70 early provitamin A-QPM inbred lines using the UPGMA applied to Nei’s 1983 genetic distance generated from the DArTseq markers

Based on the Nei’s genetic distance, the UPGMA phylogenetic tree displayed five main groups for the 70 inbred lines (Fig. 2). Thirteen inbred lines constituted group I, group II had 8, group III consisted of 18, group IV had 6 while group V was made up of 25 inbred lines. With the exception of the six checks, all the inbred lines had been improved through direct selection for *Striga* resistance and drought tolerance, indirect selection for low-N tolerance as well as direct selection for increased PVA and quality protein levels. The six checks (two normal yellow and four QPM yellow endosperm inbred lines) constituted group IV. Available information on the inbred lines revealed that the different groups and sub-groups largely depended on pedigree information, the presence and the dose of genes for drought tolerance, as well as PVA and quality protein (lysine and/ or tryptophan) levels.

### Population structure and principal component analyses

**Fig. 3:**
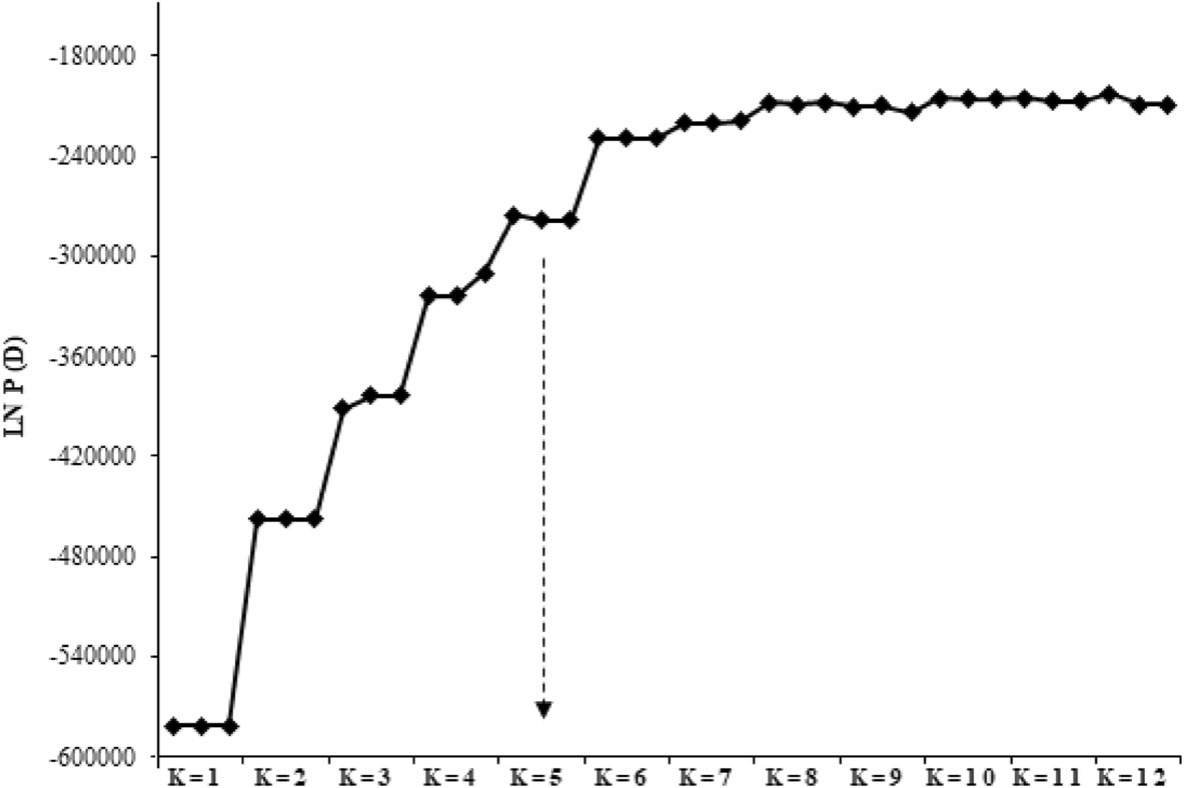
Assessment of the best K in structure analysis using changing trends of estimated Ln probability of data LnP (D) over three repeats at each K value

**Fig. 4:**
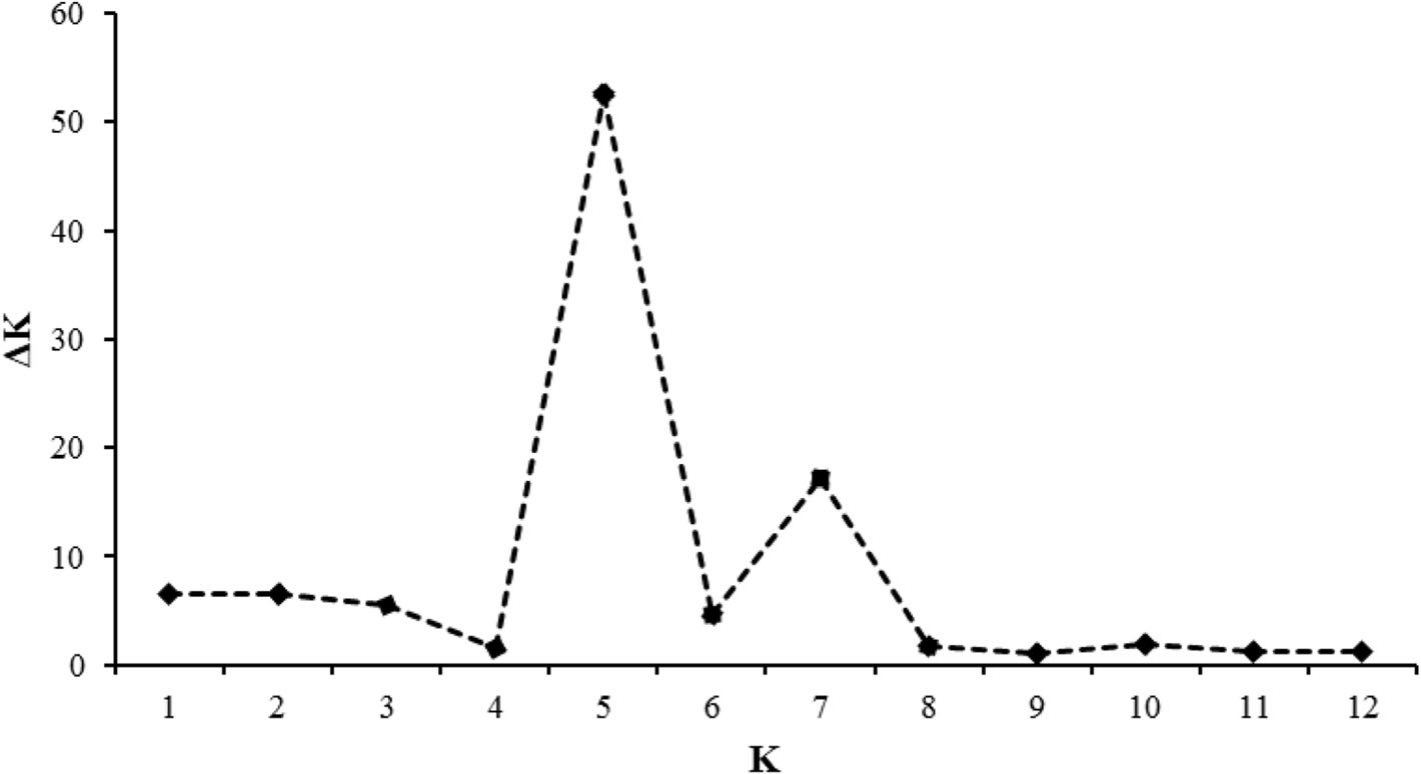
Assessment of the best K in structure analysis using and Pritchard’s K (ΔK)

**Fig. 5:**
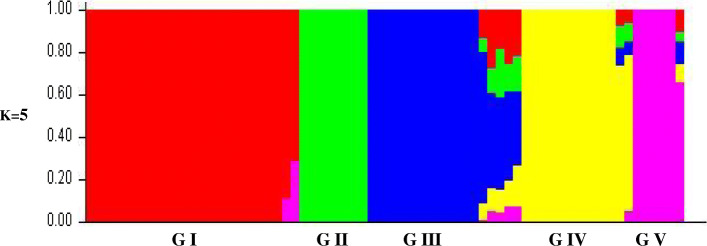
Population structure bar plot of the 70 early PVA-QPM inbred lines as membership coefficients (Q values). 25 inbreds in group I, 8 in group II, 14 in group III, 13 in group IV, 6 in group V and 4 in mixed group

The model-based clustering using an admixture programme in the STRUCTURE software was employed to deduce the number of clusters (K) within the 70 early maturing inbred lines. In structure analysis, two criteria can be used to determine the best K in a population. These are the use of log of likelihood for each K [13, 14] and the use of an ad hoc quantity (ΔK) [9]. For the log of likelihood criterion, LnP(D) plateaued when K approached a true value (Fig. 3). On the other hand, the ΔK revealed the highest peak at the true K (Fig. 4). The two plots consistently identified five clusters in the population (Fig. 5). Sixty-six inbred lines which had probabilities ≥0.70 were allocated to a single cluster, while 4 inbreds (5.71% of the total) had probabilities < 0.70 and could not be distinctly classified into any of the groups (Table S1). The four inbreds were referred to as a mixed group. The number and the order of grouping of the inbred lines in the structure analysis were very similar to that of the UPGMA phylogeny. In the STRUCTURE bar plot, the number of inbred lines classified into each cluster varied from 25 in group I, 8 in group II, 14 in group III, 13 in group IV, 6 in group V and 4 in mixed group.

**Fig. 6:**
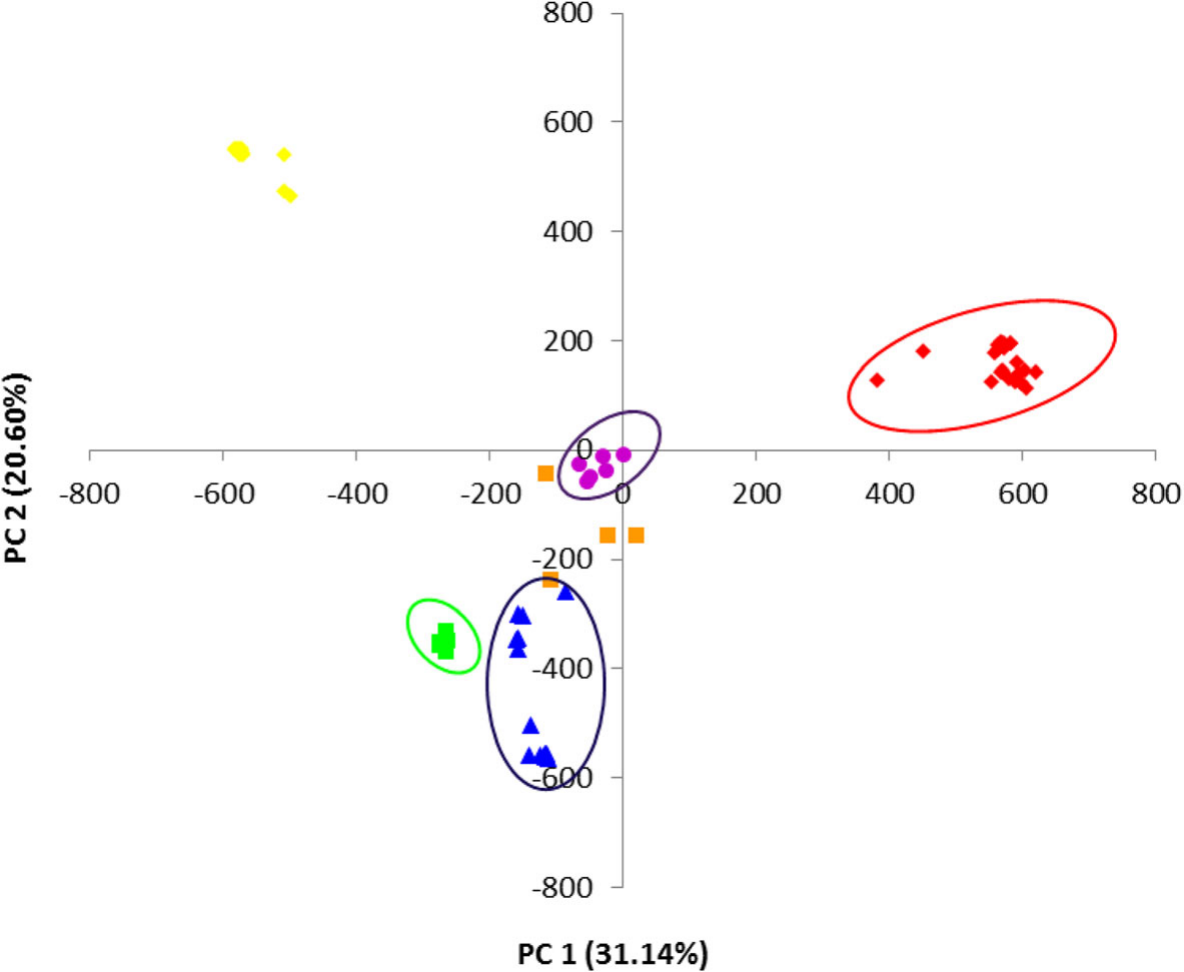
Principal Coordinate Analysis (PCoA) for the 70 early maturing provitamin A- quality protein maize inbred lines. Similar groups as identified by the structure analysis; 25 inbreds in group I, 8 in group II, 14 in group III, 13 in group IV, 6 in group V and 4 in mixed group

Principal Coordinate Analysis (PCoA) of the DArTseq data was carried out to alternatively study the structure of the inbred population [15]. The output of the PCoA was highly consistent with that of the structure analysis. As illustrated in Fig. 6, the PCoA clearly revealed 5 groups of inbred lines similar to those identified in the structure analysis.

### Analysis of variance of hybrids for agronomic traits

Mean squares of environment (E), hybrid (G), and their interactions (GEI) were significant (*p* < 0.01 or 0.05) for measured traits under each and across environments except mean squares of GEI for ASI under low-N, and grain yield across environments (Tables S2 and S3). Mean squares for research condition (Rcond) × hybrid interactions showed significant (*p <* 0.01) difference only for ASI. GCA male, GCA female, SCA, GCA male × E, GCA female × E, and SCA × E revealed significant (*p <* 0.01 or 0.05) differences for all traits. The few exceptions were GCA male × E interactions for ASI under optimal conditions and across environments, and GCA female × E for ASI under optimal conditions. Comparison of grain yield performance of the hybrids under low-N and optimal conditions revealed yield reductions ranging from 10% for TZEIORQ 29 × TZEIORQ 43 to 71% for TZEIORQ 43 × TZEIORQ 41, with a mean of 34% (Table 1). The top performing hybrid, TZEIORQ 40 × TZEIORQ 26 (based on the multiple trait index under low-N) out-yielded the best commercial hybrid control, TZEIOR 127 × TZEIOR 57 by 8, 3, and 9% under low-N, optimal, and across environments, respectively. Among the top performing hybrids under low-N conditions, only TZEIORQ 29 × TZEIORQ 43 significantly (*p* < 0.05) yielded better than the best commercial hybrid control. However, the yield was not significantly different from those of the top 15 hybrids.

**Table 1:** Grain yield and other agronomic traits of 20 early provitamin A- quality protein maize hybrids (best 15 and worst 5) and four checks evaluated under low-N and optimal environments in 2016 and 2017 growing seasons at Ile-Ife and Mokwa, Nigeria

HYBRID	Grain Yield (kg/ha)			% YRD	Days to 50% Silking		Plant Aspect		Ear Aspect		Ears per plant		STGR	MI_LN
	Low-N	Optimal	Across Env		Low-N	Optimal	Low-N	Optimal	Low-N	Optimal	Low-N	Optimal		
TZEIORQ 40 x TZEIORQ 26	4155	5911	5207	29	52	51	4	3	4	2	0.92	0.95	2	6.18
TZEIORQ 42 x TZEIORQ 20	4584	5905	5245	22	52	52	4	3	3	3	0.83	0.93	3	5.76
TZEIORQ 43 x TZEIORQ 5	4359	6101	5230	29	51	50	5	4	4	3	0.91	0.96	2	5.44
TZEIORQ 47 x TZEIORQ 15	4004	5663	4833	29	52	51	4	3	4	3	0.96	0.98	3	5.29
TZEIORQ 29 x TZEIORQ 43	5055	5639	5458	10	54	53	5	4	4	3	0.76	0.96	2	5.11
TZEIORQ 23 x TZEIORQ 11	4623	5729	5392	19	53	51	4	3	4	3	0.79	0.92	3	4.70
TZEIORQ 26 x TZEIORQ 47	4160	5857	5109	29	52	52	4	3	4	3	0.85	0.85	3	4.59
TZEIORQ 40 x TZEIORQ 2	4556	6082	5319	25	53	51	4	3	4	3	0.91	0.97	4	4.58
TZEIORQ 7 x TZEIORQ 42	4016	5655	4886	29	52	51	5	4	4	3	0.97	0.97	3	4.46
TZEIORQ 26 x TZEIORQ 13	4384	6198	5341	29	53	52	4	3	4	3	0.92	0.95	4	4.43
TZEIORQ 70 x TZEIORQ 2	4369	6016	5592	27	54	54	5	4	4	3	0.91	0.99	3	4.43
TZEIORQ 42 x TZEIORQ 11	4432	5530	4981	20	52	51	5	4	4	3	0.8	0.96	3	3.61
TZEIORQ 43 x TZEIORQ 26	4030	5620	4925	28	52	50	5	4	4	2	0.86	0.87	3	3.58
TZEIORQ 48 x TZEIORQ 43	4694	5888	5291	20	53	52	5	4	4	3	0.75	0.97	3	3.54
TZEIORQ 43 x TZEIORQ 2	4440	6074	5507	27	52	51	5	4	4	3	0.91	0.98	4	3.50
TZEIORQ 7 x TZEIORQ 23	1444	2537	1990	43	57	54	7	6	7	5	0.59	0.66	5	−8.24
TZEIORQ 41 x TZEIORQ 47	933	2934	1933	68	54	53	7	6	7	5	0.67	0.85	5	−10.38
TZEIORQ 23 x TZEIORQ 20	935	2495	1715	63	56	55	7	6	7	5	0.64	0.66	5	−10.63
TZEIORQ 40 x TZEIORQ 41	932	2401	1657	61	55	54	7	6	7	6	0.55	0.71	5	−11.37
TZEIORQ 43 x TZEIORQ 41	747	2568	1657	71	57	55	7	6	7	5	0.62	0.71	5	−13.17
C1-TZEIOR 127 x TZEIOR 57	3805	5712	4758	33	53	51	5	4	4	3	0.92	0.92	3	3.78
C2-TZEI 124 x TZEI 25	3690	6074	4882	39	55	53	4	3	4	3	0.78	0.88	3	3.41
C3-TZE Pop DT STR x TZEI 17	2962	5500	4231	46	54	52	5	4	5	4	0.77	0.83	3	0.67
C4-TZE Pop DT STR x TZEI 13	2430	4381	3406	45	56	54	5	4	5	4	0.7	0.73	4	−1.62
MEAN	3542.60	5040.15	4363.44	34.03	53.30	52.06	5.15	4.15	4.70	3.45	0.81	0.89	3.50	
SED	533.27	585.01	395.80	–	0.67	0.64	0.39	0.37	0.46	0.48	0.08	0.09	0.41	
**REPEATABILITY (R)**	**0.75**	**0.82**	**0.80**	**–**	**0.81**	**0.85**	**0.78**	**0.86**	**0.81**	**0.88**	**0.47**	**0.55**	**0.72**	

*Across Env* across low-N and optimal environments, *%YRD* percentage yield reduction, *PASP* plant aspect (1–9), *EASP* ear aspect (1–9), *EPP* ears per plant, *STGR* stay green characteristic, *MI_LN* multiple trait base index under low-N, *C1 to C4* checks 1 to 4 respectively

### Estimates of repeatability and breeding efficiency

Repeatability (R) values were relatively higher under optimal environments compared to that of low-N (Table 1). High R estimates were detected for grain yield and most other traits under both low-N and optimal conditions. Similarly, high breeding efficiency of 71, 70 and 72% were computed under low-N, optimal, and across environments respectively (Table 2). Over 97% of the crosses classified as the 32 high yielding inter-group hybrids met the set criterion, while more than 84% of the crosses categorized as the 32 low yielding intra-group hybrids also satisfied that criterion for the estimation of breeding efficiency under low-N, optimal and across research conditions.

**Table 2:** Breeding efficiency of the five clusters identified by the DArTseq markers under low- N, optimal and across environments

Yield Group	Cross type	DArTseq Markers
**Low N environments**		
1	Inter	31
1	Intra	1
2	Inter	32
2	Intra	0
3	Inter	4
3	Intra	28
**Breeding Efficiency (%)**		**71.41**
**Optimal environments**		
1	Inter	31
1	Intra	1
2	Inter	31
2	Intra	1
3	Inter	5
3	Intra	27
**Breeding Efficiency (%)**		**69.69**
**Across environments**		
1	Inter	32
1	Intra	0
2	Inter	31
2	Intra	1
3	Inter	4
3	Intra	28
**Breeding Efficiency (%)**		**72.16**

## Discussion

The degree of genetic dissimilarity and population structure of the 70 early maturing inbred lines were assessed using 8171 DArTseq markers. The summary statistics, UPGMA phylogeny, structure analysis, and PCoA were used to investigate the inbred population to ascertain whether the population was homogeneous or harboured genetically distinct groups. The average gene diversity (GD) of 0.36 obtained was higher than the 0.35 reported by Zhang et al. [4] when a tropical group of maize varieties was studied. The GD was also higher than that obtained in previous studies by Lu et al. [16] and Van Inghelandt [17] which was around 0.32, but was lower than the value reported by Wu et al. [18] which was 0.364, as well as that obtained by Yang et al. [19] which was 0.39. The results agreed with previous findings that tropical maize germplasm is highly diverse with GD > 0.3 [4, 20]. Additionally, the observed average residual heterozygosity of 5.6% was higher compared with the 3.80% obtained by Dao et al. [5], as well as the 3.34% found by Liu et al. [21], but was lower than the 8.6% obtained by Jambrovic et al. [22]. The practically acceptable level of average heterozygosity indicated that the proportion of heterozygous individuals in the set of inbred lines was reasonably low with most of the inbreds being about 94.4% homozygous with their loci fixed with minimal segregation. The average PIC obtained, 0.289 (ranging from 0.041 to 0.375) using 8171 SNPs for the 70 inbred lines was higher than the 0.24 reported by Yu et al. [23] using 1000 SNPs for a sample size of 274, as well as the 0.256 reported by Dao et al. [5] using 1057 SNPs for a sample size of 100. This result indicated the existence of a high frequency of alleles and therefore high genetic diversity in the population as evident in the average major allele frequency of 0.74. The average PIC value was similar to that reported by Wu et al. [18] which was 0.29 using varying number of SNPs up to 43,252 for a sample of 1857. The differences in the results of this study relative to other studies may be due to the use of different genetic materials, the sample sizes and the number of SNPs used.

Based on the 8171 SNPs and the Nei’s [24] genetic distance methods, the UPGMA phylogeny using 1000 non-parametric bootstrapping revealed five clusters for the 70 inbred lines. The clustering together of particular inbred lines into a group illustrated that the DArTseq markers were identical in state at common loci for those inbred lines and that there was the tendency for such inbred lines to be more related than those in different groups. The inbred lines were extracted from the source population 2009 TZE - OR2 DT STR QPM which is early maturing, has elevated levels of PVA and quality protein, drought tolerance (and tolerance to low-N through unintentional selection) and *Striga* resistance [25, 26]. The 13 inbred lines in group I followed pedigree information or were identical by descent, and were mostly drought and moderately low-N tolerant. The eight inbred lines in group II were related by pedigree records and were mostly drought tolerant. Group III consisted of 18 inbred lines which had the PVA background coupled with low-N tolerance. Inbred lines in group III including TZEIORQ 10, TZEIORQ 12, TZEIORQ 13, TZEIORQ 14, TZEIORQ 15, TZEIORQ 16 and TZEIORQ 17 had the functional PVA gene (crtRB1) [26]. Also, in group III, inbred lines consisting of TZEIORQ 55, TZEIORQ 29 and TZEIORQ 20, contained moderate to high PVA contents. It was striking that all the six inbred checks were classified into group IV. The inbred checks had yellow kernels and mostly possessed high quality protein levels and hence their clustering might have been influenced by common loci responsible for these traits. Group V was relatively larger and contained inbred lines which were related by descent [25, 26].

The log of likelihood method of determining the best K showed a steep increase in LnP(D) values from K = 1 to K = 5 after which the trend assumed a plateau. This observation indicated that the true K was 5 [13, 14] and that five genetically distinct clusters existed in the entire population. Similarly, the Evanno criterion identified the peak level of ΔK at K = 5 [14] inferring five genetically distinct clusters. The three multivariate analyses illustrated the existence of genetically different groups among the inbred lines. Comparison of the different methods revealed high consistency among the UPGMA clustering, the structure analysis, and the PCoA clustering considering the number of groups and number of individuals assigned to each group indicating that the identified groups were indeed genetically distinct. Individuals from the different groups were therefore, expected to harbour different favourable alleles for breeding for drought and/ low-N tolerant hybrids and synthetics with elevated PVA and QPM contents. The result contradicted the findings of Dao et al. [5] and Semagn et al. [3] who reported a high consistency among structure analysis and the PCoA but a low concordance with the neighbour-joining phylogeny generated using the Roger’s genetic distance method. This could be due to the differences in the inbred lines used in the different studies, the different methods of obtaining the genetic distances among the inbreds, as well as the different clustering algorithms. The clustering according to the three multivariate analyses employed in the present study followed the available pedigree information, that is, expected related lines clustered together. This result substantiated other reports that the grouping of the tropical maize populations were largely consistent with pedigree records [3, 5, 27]. The result also demonstrated that the SNP-based DArT derived markers were informative in providing genome profiles which are very useful for the identification of unique characteristics among the inbred lines [3, 28].

The highly significant mean squares of G and E observed for most measured traits including grain yield and stay green characteristic under low-N, optimal, and across environments implied the existence of high genetic variability among the 96 hybrids generated [29] and that the test environments were unique and effectively revealed genetic differences among the hybrids to warrant selection. The significant mean squares of GEI detected for grain yield and most measured traits under each and across research conditions indicated that environmental variation controlled the expression of traits thus substantiating the need to conduct genotype evaluations across multiple environments [30–32] to better assess grain yield performance and stability of genotypes [33, 34]. The significant GCA male, GCA female, SCA, and their interactions with environments for grain yield and most other traits suggested that additive and non-additive genetic effects controlled the inheritance of the traits. This implied that good parental inbreds could be identified for further improvement in the traits while outstanding hybrids could be selected for commercialization. The range of grain yield reduction (10–71%) observed for the hybrids was higher compared to the 10–50% reported by [7]. This suggested that the low-N conditions imposed were more severe and therefore, identified top performing hybrids were likely to possess low-N tolerant genes which might have been inherited from the parental inbreds [10]. The top-ranking hybrid, TZEIORQ 29 × TZEIORQ 43 which significantly out-yielded the controls under low-N with non-significant yield penalties under favourable growing conditions was also not significantly different from the remaining 14 top-performing hybrids. This suggested that all the 15 top-performing hybrids should be further evaluated to confirm consistency of performance under each and across environments for release and commercialization. These hybrids would be the hybrids of choice because they possess better grain yield potential compared to the four hybrid controls, TZEIOR 127 × TZEIOR 57, TZEI 124 × TZEI 25, TZE Pop DT STR × TZEI 17, and TZE Pop DT STR × TZEI 13 which have been released and commercialized (except TZEIOR 127 × TZEIOR 57) in Nigeria, Ghana and Mali [25]. Moreover, the promising hybrids in the pipeline have extra advantage of elevated levels of PVA, lysine and tryptophan which are lacking in the available commercial hybrid checks.

The high repeatability estimates detected for grain yield and most other traits under each and across environments implied that repeated evaluations of the hybrids under the different research conditions would yield results consistent with those of the present study. Thus, genetic effects were preponderance over environmental effects to modulate the expression of the measured traits. The results also suggested that direct selection for grain yield under each and across environments would be effective. Contrary to this result, several authors have obtained low repeatability estimates for grain yield particularly under stress environments partly due to the quantitative nature of the inheritance of the trait, and the severity of the stress imposed [35–38]. The differences in the results could be due to the different genetic materials, and level of stress imposed. It was striking to detect high breeding efficiency under low-N, optimal and across environments indicating that the inbred lines involved in the hybrid combinations belonged to genetically distinct groups as revealed by the results of the molecular (DArTseq) marker analysis. Similar results were found by [39] who detected high breeding efficiency for hybrids using SNP marker groupings of early white inbred lines. The results suggested that maximum heterosis (more productive crosses) could be exploited from the early PVA-QPM inbred set under low-N, and optimal conditions by selecting parental lines from opposing groups generated by the DArTseq markers.

## Conclusions

The clustering according to admixture implemented in the structure software, the principal Coordinate Analysis (PCoA) of the DArTseq markers and the UPGMA phylogeny consistently revealed five groups each which followed pedigree records. The three analyses were also highly consistent regarding the number of inbreds assigned to a group. Closely related genotypes with common characteristics including high PVA, lysine and tryptophan contents, and also drought and low-N tolerance were assigned to common groups indicating the existence of genetically distinctiveness between groups in the set of inbreds assessed. Hybrid evaluations showed high breeding efficiency under low-N, optimal and across environments indicating that the inbred lines involved in the hybrid combinations belonged to genetically distinct groups as revealed by the molecular (DArTseq) markers. The 15 top performing hybrids identified out-yielded the four hybrid controls under low-N conditions and suffered no yield penalties under favourable growing conditions. The hybrids should be further evaluated to confirm consistency of performance under each and across environments for release and commercialization. The inbred lines from opposing clusters could therefore be exploited for developing drought and/ or low-N tolerant hybrids and synthetics with elevated PVA and quality protein contents for commercialization in SSA, and for the improvement of the early maturing PVA-QPM inbred lines.

## Methods

Seventy early maturing PVA-QPM inbred lines recently developed by the IITA-maize improvement programme were used in the current study (Table S4). All genetic materials were sourced from the IITA maize programme. The development of the lines commenced in 2007 and by 2015 they were at the S_7_ generation of inbreeding as described by Badu-Apraku and Fakorede [25] and Obeng-Bio et al. [26]. Briefly, the lines were generated from the 2009 TZE-OR2 DT STR QPM variety which was formed from a BC_1_F_3_ generation derived from the cross between the *Striga* resistant and drought tolerant early QPM orange/yellow population, TZE-Y Pop DT STR QPM and [Syn-KU1409/DES/1409 (OR2)], an intermediate variety (105–110 days to physiological maturity) with high PVA content. During the inbreeding programme, the inbred lines were evaluated from the S_2_ to the S_5_ generations to identify those with deep orange kernel colour and possessing 25–50% opaqueness. The present study assessed the genetic diversity of the 70 IITA inbred lines including 66 PVA-QPM lines along with two normal yellow and two yellow QPM inbred checks, also from the IITA maize improvement programme. The checks were selected based on their reactions to drought and low-N stresses. In addition, 24 inbred lines were selected based on the kernel colour for provitamin A levels, endosperm opaqueness (25–50%) for tryptophan and lysine content, and tolerance to low-N and drought [25, 26]. These were used to develop 96 single crosses utilizing the North Carolina II mating design. The 96 hybrids were subsequently tested under low-N and optimal environments at Mokwa and Ile-Ife during the 2016 and 2017 growing seasons in Nigeria.

### Collection of leaf samples and DNA extraction

Leaves of the inbred lines were sampled from 10 typical plants (one leaf per plant) of each inbred line at 2 weeks after planting (WAP). The leaf samples were freeze-dried, and genomic DNA samples were extracted from the leaf tissues following the DArT DNA extraction protocol [40]. DNA concentration of 30 ng/ μl was obtained for each sample (Thermo Scientific, USA). The quality of DNA was determined on 0.8% agarose gel and short or degenerated DNA were discarded.

### Diversity Array technology sequencing (DArTseq) genotyping

Genotyping by sequencing was performed for the PVA-QPM inbred lines with a high-density whole-genome profiling of DArT services [11]. The genotyping services were provided by the Integrated Genomic Service and Support (IGSS) platform of BecA-ILRI in Kenya deploying 44,391 DArTseq codominant markers. Data generated were analysed with the DArTsoft (DArT P/L, Canberra, Australia) software as described by DArT Pty Ltd., Australia [40].

### Evaluation of hybrids

The 96 single crosses and four commercial hybrid checks were tested under low-N (30 kg/ ha) conditions at Ile-Ife (7° 28′ N, 4° 33′ E, and 244 m above sea level, 1200 mm annual rainfall) and Mokwa (9^о^18’N, 5^о^ 4′E, 457 m altitude, 1100 mm annual rainfall) in the 2016 and 2017 growing seasons. The soil types at Ile-Ife and Mokwa are Alfisol and Luvisol, respectively [41]. Before the establishment of the low-N trials, the fields were continuously used for high density maize cultivation without N fertilizer application for several years and the biomass was completely removed from the field immediately after each harvest. These measures were adopted to deplete the soil of N. Thereafter, the nitrogen (N), phosphorus (P) and potassium (K) levels of the soil from each location were determined from the depth of 0 to 15 cm following the Kjeldahl digestion and colorimetric method [42] at the IITA analytical services laboratory, Ibadan, Nigeria. The soil from the low-N field at Ile-Ife had 0.084 g/kg of N, 2.05 g/kg of P and 0.358 g/kg of K, while that of Mokwa contained 0.085 g/kg of N, 6.32 g/kg of P and 0.20 g/kg of K. From the soil test, NPK-fertilizer that contained urea, single-superphosphate and muriate-of-potash was formulated and applied immediately after thinning. This brought the levels of the total available basal N to 15 kg/ha while the P_2_O_5_ and K_2_O levels provided 60 kg/ha each of P and K. At 4 WAP, 15 kg/ha of urea was applied to obtain 30 kg/ha of total N. The hybrids were also evaluated under optimal conditions at Ile-Ife and Mokwa during the 2016 and 2017 growing seasons. For the experiments under optimal conditions, N P K (15:15:15) was applied at 2 WAP to supply 60 kg/ha each of N, P and K and top-dressed with an additional 30 kg/ha of N at 4 WAP. A 10 × 10 alpha lattice design with two replicates was used in both low-N and optimal experiments. An experimental unit consisted of a single-row plot, 4 m long with row and hill spacings of 0.75 and 0.40 m, respectively. Two stands per hill were maintained to obtain about 66,666 plants per hectare. Pre- and post- emergence herbicides with the active ingredients primextra and paraquat respectively, were applied at the rate of 5 l/ha to suppress weed growth. Manual weeding was also done intermittently to ensure effective weed control.

### Agronomic data collected

The number of days to 50% anthesis (DA) and silking (DS), and plant height (PLHT) were recorded. Plant aspect (PASP) scores were obtained using a scale of 1–9, where 1 denoted excellent overall appearance of plants and 9 extremely poor overall appearance of plants. Ear aspect (EASP) was also rated on a 1–9 scale, where 1 indicated well-filled ears with no insect and disease damages and 9 represented plots with ears having only one or no kernel. Anthesis–silking interval (ASI) was computed as the difference between DA and DS, while the number of ears per plant (EPP) was derived as the number of ears harvested per plot divided by the number of plants in the same plot. Stay-green characteristic (STGR) was rated under low-N at 70 days after planting (DAP), that is, soft dough development stage [43], on a 1–9 scale where 1 = less than 0.10 dead leaf area, and 9 = more than 0.80 dead leaf area. Grain yield (kg/ha) was calculated using the grain weight adjusted to 15% moisture content under low-N conditions. However, under optimal conditions, a shelling percentage of 80 was assumed per plot for the hybrids and grain yield (kg/ha) was calculated using ear weight adjusted to 15% moisture content.

### Statistical analysis

DArTseq markers with > 80% call rate were retained prior to statistical analysis. Thereafter, markers with > 10% missing rate were filtered out using the TASSEL software version 5.2.12 [44]. Minimum and maximum frequencies of 0.05 and 0.95 respectively, were also considered for the filtering of markers to finally retain 8171 markers for all subsequent analyses. Summary statistics including gene diversity, heterozygosity, polymorphic information content (PIC) and major allele frequency were computed with PowerMarker version 3.25 [45]. Gene frequency, and frequency based genetic distance matrix consisting of the 70 inbred lines were estimated for the DArT-seq data using the Nei [24] method implemented in PowerMarker version 3.25. Using the frequency based genetic distance estimates, the Unweighted Pair Group Method with Arithmetic Mean (UPGMA) and 1000 nonparametric bootstrapping across different loci were applied in PowerMarker to construct a dendrogram to visualize patterns of genetic dissimilarities in the panel of 70 lines.

In order to determine the genetic structure of the inbreds the STRUCTURE software package version 2.3.4 [46] which implements a Bayesian clustering procedure was used to analyse the 8171 DArTseq data. The number of sub-groups (K) was determined using a procedure that implemented shared allele frequencies and admixture. The number of K was set to vary from 1 to 12 with 10 replications. Each replication was programmed to run for 10,000 burn-period and 100,000 Markov Chain Monte Carlo (MCMC) iterations. The Log of likelihood [LnP(D)] in STRUCTURE analysis and the derived change in K (ΔK) were used to predict the true K value online from the STRUCTURE Harvester [14]. The derived ΔK takes into account the changing trend of LnP(D) with increase in K, as well as the variance of LnP(D) as the runs are repeated. The ΔK reaches the highest peak when the true value of K is realized. The formula used was:

$$ \Delta \mathrm{K}=\mathrm{M}\left[\left|\mathrm{L}\left(\mathrm{K}-1\right)-2\mathrm{L}\left(\mathrm{K}\right)+\mathrm{L}\left(\mathrm{K}+1\right)\right|\right]/\mathrm{S}\left[\mathrm{L}\left(\mathrm{K}\right)\right] $$

where: L(K) is the K^th^ LnP(D), M is the mean of 10 runs, and S, the standard deviation. The K with the maximum likelihood was identified as the true K and was used to classify the inbred lines into groups. Individual inbreds with membership probability greater than or equal to 0.70 were classified into the same group while inbreds with membership probability less than 0.70 constituted a mixed group [16, 19]. Principal Coordinate Analysis (PCoA) of the DArTseq markers was performed using GenALEx version 6.5 [47, 48].

For the hybrid trials, location by year combinations constituted an environment while the low-N and optimal environments represented research conditions. Analysis of variance (ANOVA) based on the NCII mating arrangement was performed on plot mean basis for the agronomic data under each and across research conditions using the general linear model procedure (PROC GLM) in SAS, version 9.4 [49]. The model was fitted with environments, replicates within environments, and incomplete blocks within replicates × environment interaction as random factors while the hybrids were fixed. The lattice design [50] allowed block effects on means of hybrids to be adjusted and differences among means were separated using standard error of difference (S.E.D.). The NCII design partitioned the variation among hybrids into male within sets, female within sets and male × female interaction within sets. General and specific combining ability (GCA, SCA) effects were estimated as described by Hallauer and Miranda [51]. Genetic and phenotypic variance components of the inbreds were estimated using the restricted maximum likelihood (REML) method and with PROC Varcomp implemented in SAS, repeatability (R) for each of the measured traits was estimated [52].

Low-N tolerant hybrids were identified using the multiple trait base index under low-N conditions as described by Badu-Apraku et al. [38] as follows:

MI = [(2 x Grain yield) + EPP – ASI – PASP – EASP – STGR].

where MI = multiple trait base index, EPP = number of ears per plant, ASI = anthesis-silking interval, PASP = plant aspect, EASP = ear aspect, STGR = stay green characteristic.

The traits employed in the MI were standardized to reduce the effects of unequal scales with positive and negative values indicating tolerance and susceptibility to low-N, respectively.

Breeding efficiency (B.E) was calculated for the parental lines on the basis of the groups revealed by the DArTseq markers under low-N, optimal, and across research conditions. The 96 hybrids were ranked from the highest to the lowest using grain yield under each and across environments. Estimation of B. E for each research condition involved dividing the total number of hybrids into inter-group and intra-group crosses as described by Badu-Apraku et al. [39]. The relationship among high yielding intergroup hybrids and total number of intergroup hybrids as well as the low yielding intragroup hybrids and total number of intragroup hybrids [39] was used to compute B. E as follows:

$$ BE=\frac{\left[\frac{HYINTERGH}{TNINTERGH}\times 100\right]+\left[\frac{LYINTRAGH}{TNINTRAGH}\times 100\right]}{2} $$

Where, HYINTERGH = number of high yielding inter-group hybrids, TNINTERGH = total number of inter-group hybrids, LYINTRAGH = number of low yielding intra-group hybrids, and TNINTRAGH = total number of intra-group hybrids. Efficient and productive crosses allowed inter-group crosses to produce more superior hybrids than the intra-group crosses [53].

## Supplementary information

**Additional file 1: Table S1.** Designations and pedigrees of the 64 early provitamin A quality protein maize inbreds plus six checks. **Table S2.** Probabilities for assigning an individual inbred line into a group as determined by the model-based structure analysis. **Table S3.** Mean squares of grain yield and other agronomic traits of early maturing provitamin A - quality protein maize hybrids evaluated under low-N and optimal environments at Ile-Ife and Mokwa in Nigeria during the 2016 and 2017 growing seasons. **Table S4.** Mean squares of grain yield and other agronomic traits of early maturing provitamin A - quality protein maize hybrids across low-N and optimal environments at Ile-Ife and Mokwa in Nigeria during the 2016 and 2017 growing seasons.

## Data Availability

The datasets used and/or analysed for the present study are available to authorized users at the International Institute of Tropical Agriculture (IITA) maize improvement programme data repository as follows: Phenotypic data of 70 early PVA-QPM maize hybrids evaluated under optimal conditions at Ile Ife and Mokwa, 2016 & 2017. DOI: 10.25502/tenz-sh65/d Phenotypic data of 70 early PVA-QPM maize hybrids evaluated under low N conditions at Ile Ife and Mokwa, 2016 & 2017. DOI: 10.25502/6r18-0206/d Genotypic data of 70 early maturing PVA-QPM maize lines for diversity study. DOI: 10.25502/m5e9-yc02/d

## Abbreviations

DArTseq: Diversity Array Technology sequencing
GD: Gene diversity
IGSS: Integrated Genomic Service and Support
IITA-MIP: International Institute of Tropical Agriculture Maze Improvement Programme
NGS: Next Generation Sequencing
PCoA: Principal Component Analysis
PIC: Polymorphic information content
PVA: Provitamin A
QPM: Quality protein maize
SNP: Single Nucleotide Polymorphism
UPGMA: Unweighted Pair Group Method with Arithmetic Mean
DA: Days to 50% anthesis
DS: Days to 50% silking
PLHT: Plant height
PASP: Plant aspect
EASP: Ear aspect
ASI: Anthesis silking interval
STGR: Stay green characteristic
DAP: Days after planting
WAP: Weeks after planting
GCA: General combining ability
SCA: Specific combining ability
REML: Restricted maximum likelihood
MI: Multiple trait base index
BE: Breeding efficiency
HYINTERGH: High yielding inter-group hybrids
TNINTERGH: Total inter-group hybrids
LYINTRAGH: Low yielding intra-group hybrids
TNINTRAGH: Total intra-group hybrids
