# Supplementary material for: Genetic diversity among early provitamin A quality protein maize inbred lines and the performance of derived hybrids under contrasting nitrogen environments
Source: BMC Genet. 2020 Jul 18;21:78. doi: 10.1186/s12863-020-00887-7 (PMC7368723; doi:10.1186/s12863-020-00887-7)
Supplement: Supplementary file 1 — Additional file 1: Table S1. Designations and pedigrees of the 64 early provitamin A quality protein maize inbreds plus six checks. Table S2. Probabilities for assigning an individual inbred line into a group as determined by the model-based structure analysis. Table S3. Mean squares of grain yield and other agronomic traits of early maturing provitamin A - quality protein maize hybrids evaluated under low-N and optimal environments at Ile-Ife and Mokwa in Nigeria during the 2016 and 2017 growing seasons. Table S4. Mean squares of grain yield and other agronomic traits of early maturing provitamin A - quality protein maize hybrids across low-N and optimal environments at Ile-Ife and Mokwa in Nigeria during the 2016 and 2017 growing seasons. [file 12863_2020_887_MOESM1_ESM.docx]

**Additional files**

**Table S1. Probabilities for assigning an individual inbred line into a group as determined by the model-based structure analysis**

|  | **Probabilities for clustering** | | | | |  |
| --- | --- | --- | --- | --- | --- | --- |
| **Inbred** | **Cluster I** | **Cluster II** | **Cluster III** | **Cluster IV** | **Cluster V** | **Group** |
| TZEIORQ22 | 1 | 0 | 0 | 0 | 0 | 1 |
| TZEIORQ39 | 1 | 0 | 0 | 0 | 0 | 1 |
| TZEIORQ40 | 1 | 0 | 0 | 0 | 0 | 1 |
| TZEIORQ41 | 1 | 0 | 0 | 0 | 0 | 1 |
| TZEIORQ42 | 1 | 0 | 0 | 0 | 0 | 1 |
| TZEIORQ43 | 1 | 0 | 0 | 0 | 0 | 1 |
| TZEIORQ44 | 1 | 0 | 0 | 0 | 0 | 1 |
| TZEIORQ45 | 1 | 0 | 0 | 0 | 0 | 1 |
| TZEIORQ46 | 1 | 0 | 0 | 0 | 0 | 1 |
| TZEIORQ47 | 1 | 0 | 0 | 0 | 0 | 1 |
| TZEIORQ68 | 0.737 | 0.085 | 0.065 | 0.051 | 0.063 | 1 |
| TZEIORQ69 | 0.74 | 0.103 | 0.081 | 0.001 | 0.076 | 1 |
| TZEIORQ70 | 1 | 0 | 0 | 0 | 0 | 1 |
| TZEIORQ57 | 0 | 1 | 0 | 0 | 0 | 2 |
| TZEIORQ58 | 0 | 1 | 0 | 0 | 0 | 2 |
| TZEIORQ59 | 0 | 1 | 0 | 0 | 0 | 2 |
| TZEIORQ60 | 0 | 1 | 0 | 0 | 0 | 2 |
| TZEIORQ61 | 0 | 1 | 0 | 0 | 0 | 2 |
| TZEIORQ62 | 0 | 1 | 0 | 0 | 0 | 2 |
| TZEIORQ63 | 0 | 1 | 0 | 0 | 0 | 2 |
| TZEIORQ65 | 0 | 1 | 0 | 0 | 0 | 2 |
| TZEIORQ10 | 0 | 0 | 1 | 0 | 0 | 3 |
| TZEIORQ11 | 0 | 0 | 1 | 0 | 0 | 3 |
| TZEIORQ12 | 0 | 0 | 1 | 0 | 0 | 3 |
| TZEIORQ13 | 0 | 0 | 1 | 0 | 0 | 3 |
| TZEIORQ14 | 0 | 0 | 1 | 0 | 0 | 3 |
| TZEIORQ15 | 0 | 0 | 1 | 0 | 0 | 3 |
| TZEIORQ16 | 0 | 0 | 1 | 0 | 0 | 3 |
| TZEIORQ17 | 0 | 0.001 | 0.999 | 0 | 0 | 3 |
| TZEIORQ29 | 0 | 0.001 | 0.999 | 0 | 0 | 3 |
| TZEIORQ30 | 0 | 0 | 0.999 | 0 | 0 | 3 |
| TZEIORQ31 | 0 | 0 | 1 | 0 | 0 | 3 |
| TZEIORQ32 | 0 | 0 | 1 | 0 | 0 | 3 |
| TZEIORQ33 | 0 | 0 | 1 | 0 | 0 | 3 |
| TZEIORQ34 | 0.078 | 0.066 | 0.712 | 0.015 | 0.13 | 3 |
| TZEQI85 | 0 | 0 | 0 | 1 | 0 | 4 |
| TZEQI91 | 0 | 0 | 0 | 1 | 0 | 4 |
| TZEQI74 | 0 | 0.001 | 0 | 0.999 | 0 | 4 |
| TZEQI82 | 0 | 0 | 0 | 1 | 0 | 4 |

**Table S1. Continuation**

|  | **Probabilities for clustering** | | | | |  |
| --- | --- | --- | --- | --- | --- | --- |
| **Inbred** | **Cluster I** | **Cluster II** | **Cluster III** | **Cluster IV** | **Cluster V** | **Group** |
| TZEI129 | 0.083 | 0.042 | 0.109 | 0.761 | 0.005 | 4 |
| TZEI24 | 0 | 0 | 0 | 1 | 0 | 4 |
| TZEIORQ18 | 0 | 0 | 0 | 0.002 | 0.998 | 5 |
| TZEIORQ19 | 0 | 0 | 0 | 0 | 1 | 5 |
| TZEIORQ2 | 0 | 0 | 0 | 0 | 1 | 5 |
| TZEIORQ20 | 0 | 0 | 0 | 0 | 1 | 5 |
| TZEIORQ21 | 0 | 0 | 0 | 0 | 1 | 5 |
| TZEIORQ23 | 0 | 0 | 0 | 0 | 1 | 5 |
| TZEIORQ24 | 0 | 0 | 0 | 0 | 1 | 5 |
| TZEIORQ25 | 0 | 0 | 0 | 0 | 1 | 5 |
| TZEIORQ26 | 0 | 0 | 0 | 0.29 | 0.709 | 5 |
| TZEIORQ27 | 0 | 0 | 0 | 0 | 1 | 5 |
| TZEIORQ28 | 0 | 0 | 0 | 0 | 1 | 5 |
| TZEIORQ3 | 0 | 0 | 0 | 0 | 1 | 5 |
| TZEIORQ35 | 0 | 0 | 0 | 0 | 1 | 5 |
| TZEIORQ36 | 0 | 0 | 0 | 0 | 1 | 5 |
| TZEIORQ37 | 0 | 0 | 0 | 0 | 1 | 5 |
| TZEIORQ48 | 0 | 0 | 0 | 0 | 1 | 5 |
| TZEIORQ5 | 0 | 0 | 0 | 0 | 1 | 5 |
| TZEIORQ52 | 0 | 0 | 0 | 0 | 1 | 5 |
| TZEIORQ53 | 0 | 0 | 0 | 0 | 1 | 5 |
| TZEIORQ6 | 0 | 0 | 0 | 0 | 1 | 5 |
| TZEIORQ7 | 0 | 0 | 0 | 0 | 1 | 5 |
| TZEIORQ71 | 0 | 0 | 0 | 0.113 | 0.887 | 5 |
| TZEIORQ73 | 0 | 0 | 0 | 0 | 1 | 5 |
| TZEIORQ8 | 0 | 0 | 0 | 0 | 1 | 5 |
| TZEIORQ9 | 0 | 0 | 0 | 0 | 1 | 5 |
| TZEIORQ54 | 0.197 | 0.163 | 0.347 | 0.074 | 0.219 | Mixed |
| TZEIORQ55 | 0.12 | 0.127 | 0.424 | 0.074 | 0.255 | Mixed |
| TZEIORQ66 | 0.111 | 0.113 | 0.448 | 0.052 | 0.276 | mixed |
| TZEIORQ72 | 0.111 | 0.232 | 0.433 | 0.044 | 0.18 | mixed |

**Table S2. Mean squares of grain yield and other agronomic traits of early maturing provitamin A - quality protein maize hybrids evaluated under low-N and optimal environments at Ile-Ife and Mokwa in Nigeria during the 2016 and 2017 growing seasons**

| **Source** | **DF** | **YIELD** | **DA** | **DS** | **ASI** | **PLHT** | **PASP** | **EASP** | **EPP** | **STGR** |
| --- | --- | --- | --- | --- | --- | --- | --- | --- | --- | --- |
| **Low-N conditions** |  |  |  |  |  |  |  |  |  |  |
| Env | 3 | 291853629.7** | 111.51** | 181.80** | 25.46** | 95457.86** | 35.80** | 120.78** | 895.29** | 3.77** |
| Set | 5 | 1276289.4 | 7.13** | 10.07** | 0.73 | 1217.31** | 3.29** | 2.38** | 6.06 | 0.03 |
| Env × Set | 15 | 2048451.9** | 5.09** | 4.20** | 0.39 | 297.40** | 0.29 | 1.52** | 4.93 | 0.03 |
| Rep (Env × Set) | 20 | 914232.9 | 1.03 | 0.79 | 0.26 | 163.72 | 0.36 | 0.55 | 3.52 | 0.03 |
| Block (Env × Rep) | 72 | 1475216.4** | 1.95** | 2.71** | 0.51** | 351.11** | 1.12** | 1.19** | 5.31* | 0.03 |
| Hybrid | 99 | 9948239** | 11.74** | 13.13** | 0.51* | 1316.85** | 4.83** | 6.55** | 0.09** | 3.23** |
| Male (Set) | 18 | 8473940.5** | 8.76** | 11.13** | 0.59* | 1732.69** | 3.36** | 5.72** | 10.50** | 0.10** |
| Female (Set) | 18 | 8667775.8** | 9.22** | 11.85** | 0.74** | 1627.47** | 4.63** | 6.81** | 14.70** | 0.06** |
| Female × Male (Set) | 54 | 12215059.2** | 12.98** | 13.37** | 0.42 | 1174.13** | 5.80** | 7.51** | 5.94** | 0.11** |
| Hybrid × Env | 297 | 1607731.4** | 2.30** | 2.57** | 0.39 | 253.47** | 0.63** | 0.89** | 0.04** | 0.95** |
| Env × Male (Set) | 54 | 1222434* | 1.78* | 2.09* | 0.28* | 302.00** | 0.53* | 0.8* | 4.99* | 0.04** |
| Env × Female (Set) | 54 | 1197533.8* | 2.78** | 3.23** | 0.56* | 325.76** | 0.57* | 0.7* | 6.59* | 0.03* |
| Env × Female × Male (Set) | 162 | 1805933.8** | 2.01** | 2.29** | 0.38* | 218.18** | 0.64* | 0.92** | 5.46** | 0.04** |
| Error | 288 | 862481 | 1.08 | 1.41 | 0.34 | 113.28 | 0.47 | 0.66 | 3.66 | 0.02 |
| **Optimal conditions** |  |  |  |  |  |  |  |  |  |  |
| Env | 3 | 170998881** | 458.70** | 499.79** | 4.59** | 149792.11** | 40.10** | 248.52** | 6.64** | - |
| Set | 5 | 3446360** | 6.93** | 6.13** | 0.35 | 1127.00** | 1.76** | 1.77* | 0.06* | - |
| Env × Set | 15 | 16688900 | 3.52** | 2.85** | 0.82* | 745.50** | 0.65 | 1.02 | 0.05* | - |
| Rep (Env × Set) | 20 | 1347931 | 1.12 | 1.54 | 0.42 | 191.90 | 0.60 | 1.57** | 0.02 | - |
| Block (Env × Rep) | 72 | 1520110* | 1.71** | 2.23** | 0.39 | 372.16** | 0.82** | 1.54** | 0.04** | - |
| Hybrid | 99 | 13455780** | 11.01** | 14.33** | 1.78** | 1248.58** | 3.55** | 8.10** | 0.08** | - |
| Male (Set) | 18 | 14501028** | 9.55** | 10.85** | 1.33** | 1975.63** | 4.86** | 8.73** | 0.14** | - |
| Female (Set) | 18 | 12088106** | 14.62** | 15.96** | 2.88** | 1788.85** | 3.35** | 6.24** | 0.05** | - |
| Female × Male (Set) | 54 | 15598087** | 9.87** | 15.41** | 1.68** | 898.82** | 3.80** | 9.78** | 0.07** | - |
| Hybrid × Env | 297 | 2595007** | 2.09** | 2.25** | 1.31** | 481.94** | 0.55** | 1.72** | 0.04** | - |
| Env × Male (Set) | 54 | 2371934** | 2.17** | 2.09** | 0.49 | 459.05** | 0.58* | 1.71** | 0.05** | - |
| Env × Female (Set) | 54 | 2820894** | 2.43** | 2.05** | 0.57 | 751.34** | 0.52 | 1.59** | 0.038** | - |
| Env × Female × Male (Set) | 162 | 2784931** | 1.86** | 2.36** | 0.60* | 395.49** | 0.53* | 1.91** | 0.04** | - |
| Error | 288 | 1011430 | 1.06 | 1.28 | 0.44 | 181.90 | 0.40 | 0.65 | 0.02 | - |

*, ** = Significant at 0.05 and 0.01 probability levels, respectively; Env = environment; Rep = replication; YIELD = Grain yield; DA= days to 50% anthesis ; DS = days to 50% silking; ASI = anthesis-silking interval; PLHT = plant height; PASP = plant aspect; EASP = ear aspect; EPP =ears per plant; STGR= stay-green characteristic.

**Table S3. Mean squares of grain yield and other agronomic traits of early maturing provitamin A - quality protein maize hybrids across low-N and optimal environments at Ile-Ife and Mokwa in Nigeria during the 2016 and 2017 growing seasons**

| **Source** | **DF** | **YIELD** | **DA** | **DS** | **ASI** | **PLHT** | **PASP** | **EASP** | **EPP** |
| --- | --- | --- | --- | --- | --- | --- | --- | --- | --- |
| Env | 7 | 398838807** | 333.99** | 362.84** | 14.25** | 115851.65** | 32.85** | 159.32** | 4.56** |
| Set | 5 | 2967139** | 13.17** | 14.07** | 0.7 | 1836.07** | 4.64** | 3.76** | 0.08** |
| Env × Set | 35 | 1836574** | 3.83** | 3.35** | 0.57* | 518.70** | 0.46 | 1.14** | 0.04** |
| Rep (Env × Set) | 40 | 1131082 | 1.08 | 1.16 | 0.34 | 177.81 | 0.48 | 1.06* | 0.03 |
| Block (Env × Rep) | 144 | 1497663** | 1.83** | 2.47** | 0.45 | 361.64** | 0.97** | 1.36** | 0.03** |
| Rcond | 1 | 1492985062** | 659.21** | 529.00** | 8.85** | 79521.18** | 2.33* | 8.85* | 0.64** |
| Hybrid | 99 | 22073637** | 21.20** | 26.07** | 1.33** | 2234.45** | 7.79** | 13.75** | 0.13** |
| Male (Set) | 18 | 21255279** | 16.42** | 20.30** | 1.21** | 3444.73** | 7.53** | 12.99** | 0.18** |
| Female (Set) | 18 | 19136797** | 21.59** | 26.41** | 2.03** | 3090.60** | 7.26** | 11.98** | 0.08** |
| Female × Male (Set) | 54 | 26619358** | 21.49** | 27.44** | 1.13** | 1706.86** | 9.08** | 16.47** | 0.12** |
| Rcond × Hybrid | 99 | 1544234 | 1.69 | 1.58 | 0.98** | 343.16 | 0.66 | 0.98 | 0.05 |
| Hybrid × Env | 693 | 1989799 | 2.11** | 2.27** | 0.55** | 360.38** | 0.59** | 1.25** | 0.04** |
| Env × Male (Set) | 126 | 1786544** | 1.97** | 2.03** | 0.43 | 361.85** | 0.58* | 1.29** | 0.05** |
| Env × Female (Set) | 126 | 1950409** | 2.54** | 2.45** | 0.71** | 506.12** | 0.56* | 1.13** | 0.04** |
| Env × Female × Male (Set) | 378 | 2136886** | 1.86** | 2.18** | 0.56** | 315.62** | 0.57** | 1.33** | 0.04** |
| Error | 576 | 936955 | 1.07 | 1.34 | 0.39 | 147.59 | 0.44 | 0.66 | 0.02 |

*, ** = Significant at 0.05 and 0.01 probability levels, respectively; Env = Environment; Rep = Replication; YIELD = Grain yield; DA= Days to 50% anthesis ; DS = Days to 50% silking; ASI = Anthesis-silking interval; PLHT = Plant height; PASP = Plant aspect; EASP = Ear aspect; EPP = Ears per plant; Rcond = Research condition.

**Table** **S4. Designations and pedigrees of the 64 early provitamin A quality protein maize inbreds plus the six** **checks**

|  | **Inbred-** |  | **Reactions to-** | |
| --- | --- | --- | --- | --- |
| **S/N** | **Designation-** | **Pedigree-** | **Drought-** | **Low-N-** |
| 1 | TZEIORQ-2 | 2009-TZE-OR2-DT-STR-QPM S_6_ Inb 2-2/3-1/3-1/3-1/2-1/1- | T | T |
| 2 | TZEIORQ-3 | 2009-TZE-OR2-DT-STR-QPM S_6_ Inb 2-2/3-1/3-3/3-3/3-1/1- | - | - |
| 3 | TZEIORQ-5 | 2009-TZE-OR2-DT-STR-QPM S_6_ Inb 2-2/3-2/3-1/4-3/3-1/1- | T | S |
| 4 | TZEIORQ-6 | 2009-TZE-OR2-DT-STR-QPM S_6_ Inb 2-2/3-2/3-2/4-1/5-1/1- | T | S |
| 5 | TZEIORQ-7 | 2009-TZE-OR2-DT-STR-QPM S_6_ Inb 2-2/3-2/3-3/4-1/3-1/1- | T | S |
| 6 | TZEIORQ-8 | 2009-TZE-OR2-DT-STR-QPM S_6_ Inb 2-2/3-2/3-4/4-3/3-1/1- | T | S |
| 7 | TZEIORQ-9 | 2009-TZE-OR2-DT-STR-QPM S_6_ Inb 2-2/3-3/3-1/5-1/2-1/1- | S | T |
| 8 | TZEIORQ-10 | 2009-TZE-OR2-DT-STR-QPM S_6_ Inb 7-1/3-1/2-1/2-1/4-1/1- | S | - |
| 9 | TZEIORQ-11 | 2009-TZE-OR2-DT-STR-QPM S_6_ Inb 7-1/3-1/2-1/2-4/4-1/1- | T | T |
| 10 | TZEIORQ-12 | 2009-TZE-OR2-DT-STR-QPM S_6_ Inb 7-1/3-1/2-2/2-2/3-1/1- | S | - |
| 11 | TZEIORQ-13 | 2009-TZE-OR2-DT-STR-QPM S_6_ Inb 7-1/3-1/2-2/2-3/3-1/1- | - | - |
| 12 | TZEIORQ-14 | 2009-TZE-OR2-DT-STR-QPM S_6_ Inb 7-2/3-1/2-2/4-2/2-1/1- | - | - |
| 13 | TZEIORQ-15 | 2009-TZE-OR2-DT-STR-QPM S_6_ Inb 7-2/3-1/2-3/4-1/3-1/1- | T | S |
| 14 | TZEIORQ-16 | 2009-TZE-OR2-DT-STR-QPM S_6_ Inb 7-2/3-1/2-4/4-1/3-1/1- | S | - |
| 15 | TZEIORQ-17 | 2009-TZE-OR2-DT-STR-QPM S_6_ Inb 22-1/2-1/1-2/2-3/3-1/1- | S | S |
| 16 | TZEIORQ-18 | 2009-TZE-OR2-DT-STR-QPM S_6_ Inb 22-2/2-1/3-2/3-2/2-1/1- | - | - |
| 17 | TZEIORQ-19 | 2009-TZE-OR2-DT-STR-QPM S_6_ Inb 22-2/2-1/3-3/3-1/1-1/1- | - | - |
| 18 | TZEIORQ-20 | 2009-TZE-OR2-DT-STR-QPM S_6_ Inb 26-1/1-1/2-1/6-1/2-1/1- | T | T |
| 19 | TZEIORQ-21 | 2009-TZE-OR2-DT-STR-QPM S_6_ Inb 26-1/1-1/2-3/6-1/4-1/1- | T | T |
| 20 | TZEIORQ-22 | 2009-TZE-OR2-DT-STR-QPM S_6_ Inb 26-1/1-1/2-3/6-3/4-1/1- | T | T |
| 21 | TZEIORQ-23 | 2009-TZE-OR2-DT-STR-QPM S_6_ Inb 26-1/1-1/2-4/6-1/3-1/1- | T | S |
| 22 | TZEIORQ-24 | 2009-TZE-OR2-DT-STR-QPM S_6_ Inb 26-1/1-1/2-4/6-2/3-1/1- | T | T |
| 23 | TZEIORQ-25 | 2009-TZE-OR2-DT-STR-QPM S_6_ Inb 26-1/1-1/2-5/6-1/3-1/1- | T | T |
| 24 | TZEIORQ-26 | 2009-TZE-OR2-DT-STR-QPM S_6_ Inb 26-1/1-1/2-6/6-2/3-1/1- | T | S |
| 25 | TZEIORQ-27 | 2009-TZE-OR2-DT-STR-QPM S_6_ Inb 26-1/1-2/2-3/3-1/2-1/1- | T | - |
| 26 | TZEIORQ-28 | 2009-TZE-OR2-DT-STR-QPM S_6_ Inb 28-1/1-1/2-2/2-1/3-1/1- | - | - |
| 27 | TZEIORQ-29 | 2009-TZE-OR2-DT-STR-QPM S_6_ Inb 28-1/1-2/2-1/2-1/2-1/1- | T | S |
| 28 | TZEIORQ-30 | 2009-TZE-OR2-DT-STR-QPM S_6_ Inb 28-1/1-2/2-1/2-2/2-1/1- | T | S |
| 29 | TZEIORQ-31 | 2009-TZE-OR2-DT-STR-QPM S_6_ Inb 31-1/2-1/2-2/4-1/1-1/1- | - | - |
| 30 | TZEIORQ-32 | 2009-TZE-OR2-DT-STR-QPM S_6_ Inb 31-1/2-1/2-3/4-1/1-1/1- | - | - |
| 31 | TZEIORQ-33 | 2009-TZE-OR2-DT-STR-QPM S_6_ Inb 31-1/2-4/4-1/2-1/1-1-1- | S | T |
| 32 | TZEIORQ-34 | 2009-TZE-OR2-DT-STR-QPM S_6_ Inb 31-2/2-1/2-1/2-1/2-1/1- | S | S |
| 33 | TZEIORQ-35 | 2009-TZE-OR2-DT-STR-QPM S_6_ Inb 32-1/1-1/2-1/3-2/5-1/1- | - | - |
| 34 | TZEIORQ-36 | 2009-TZE-OR2-DT-STR-QPM S_6_ Inb 32-1/1-1/2-1/3-3/3-1/1- | S | S |
| 35 | TZEIORQ-37 | 2009-TZE-OR2-DT-STR-QPM S_6_ Inb 32-1/1-1/2-2/3-2/2-1/1- | T | T |
| 36 | TZEIORQ-39 | 2009-TZE-OR2-DT-STR-QPM S_6_ Inb 35-2/3-1/3-3/5-2/3-1/1- | T | T |
| 37 | TZEIORQ-40 | 2009-TZE-OR2-DT-STR-QPM S_6_ Inb 35-2/3-2/3-1/2-2/2-1/1- | S | T |
| 38 | TZEIORQ-41 | 2009-TZE-OR2-DT-STR-QPM S_6_ Inb 35-2/3-3/3-1/4-3/3-1/1- | - | - |
| 39 | TZEIORQ-42 | 2009-TZE-OR2-DT-STR-QPM S_6_ Inb 35-2/3-3/3-2/4-2/2-1/1- | T | T |

**Table S4 Continuation**

|  | **Inbred** |  | **Reaction to-** | |
| --- | --- | --- | --- | --- |
| **S/N** | **Designation-** | **Pedigree-** | **Drought-** | **Low-N-** |
| 40 | TZEIORQ-43 | 2009-TZE-OR2-DT-STR-QPM S_6_ Inb 35-2/3-3/3-3/4-1/2-1/1- | T | S |
| 41 | TZEIORQ-44 | 2009-TZE-OR2-DT-STR-QPM S_6_ Inb 35-2/3-3/3-4/4-1/4-1/1- | T | S |
| 42 | TZEIORQ-45 | 2009-TZE-OR2-DT-STR-QPM S_6_ Inb 35-2/3-3/3-4/4-3/4-1/1- | S | T |
| 43 | TZEIORQ-46 | 2009-TZE-OR2-DT-STR-QPM S_6_ Inb 35-3/3-3/3-1/3-1/2-1/1- | - | - |
| 44 | TZEIORQ-47 | 2009-TZE-OR2-DT-STR-QPM S_6_ Inb 35-3/3-3/3-1/3-2/2-1/1- | S | T |
| 45 | TZEIORQ-48 | 2009-TZE-OR2-DT-STR-QPM S_6_ Inb 41-1/2-1/3-1/2-3/3-1/1- | S | - |
| 46 | TZEIORQ-52 | 2009-TZE-OR2-DT-STR-QPM S_6_ Inb 41-2/2-1/2-1/3-1/1-1/1- | - | - |
| 47 | TZEIORQ-53 | 2009-TZE-OR2-DT-STR-QPM S_6_ Inb 41-2/2-1/2-2/3-2/2-1/1- | S | S |
| 48 | TZEIORQ-54 | 2009-TZE-OR2-DT-STR-QPM S_6_ Inb 41-2/2-1/2-3/3-1/1-1/1- | S | T |
| 49 | TZEIORQ-55 | 2009-TZE-OR2-DT-STR-QPM S_6_ Inb 42-2/2-2/2-1/1-1/1-1/1- | - | - |
| 50 | TZEIORQ-57 | 2009-TZE-OR2-DT-STR-QPM S_6_ Inb 50-2/2-1/3-1/3-3/3-1/1- | - | - |
| 51 | TZEIORQ-58 | 2009-TZE-OR2-DT-STR-QPM S_6_ Inb 50-2/2-1/3-2/3-1/2-1/1- | S | S |
| 52 | TZEIORQ-59 | 2009-TZE-OR2-DT-STR-QPM S_6_ Inb 50-2/2-1/3-2/3-2/2-1/1- | T | T |
| 53 | TZEIORQ-60 | 2009-TZE-OR2-DT-STR-QPM S_6_ Inb 50-2/2-1/3-3/3-1/2-1/1 | - | - |
| 54 | TZEIORQ-61 | 2009-TZE-OR2-DT-STR-QPM S_6_ Inb 50-2/2-2/3-1/2-1/2-1/1- | T | T |
| 55 | TZEIORQ-62 | 2009-TZE-OR2-DT-STR-QPM S_6_ Inb 50-2/2-2/3-2/2-1/1-1/1A- | - | - |
| 56 | TZEIORQ-63 | 2009-TZE-OR2-DT-STR-QPM S_6_ Inb 50-2/2-2/3-2/2-1/1-1/1B- | - | - |
| 57 | TZEIORQ-65 | 2009-TZE-OR2-DT-STR-QPM S_6_ Inb 50-2/2-3/3-3/4-1/1-1/1- | S | S |
| 58 | TZEIORQ-66 | 2009-TZE-OR2-DT-STR-QPM S_6_ Inb 51-2/3-1/2-2/3-1/1-1/1- | S | S |
| 59 | TZEIORQ-68 | 2009-TZE-OR2-DT-STR-QPM-S_6_ Inb 57-2/2-1/2-1/4-2/2-1/1- | S | T |
| 60 | TZEIORQ-69 | 2009-TZE-OR2-DT-STR-QPM S_6_ Inb 57-2/2-2/2-1/1-1/2-1/1- | T | T |
| 61 | TZEIORQ-70 | 2009-TZE-OR2-DT-STR-QPM S_6_ Inb 60-2/2-1/2-1/3-1/4-1/1- | S | T |
| 62 | TZEIORQ-71 | 2009-TZE-OR2-DT-STR-QPM S_6_ Inb 60-2/2-1/2-2/3-1/1-1/1- | S | S |
| 63 | TZEIORQ-72 | 2009-TZE-OR2-DT-STR-QPM S_6_ Inb 62-2/2-2/2-1/2-1/2-1/1- | S | S |
| 64 | TZEIORQ-73 | 2009-TZE-OR2-DT-STR-QPM S_6_ Inb 62-2/2-2/2-2/2-1/1-1/1- | - | - |
| 65 | C-1 TZEQI-85 | TZE-COMP5-Y C6S6 Inb 25 x Pool 18 SR QPM BC2S6 4-5-1-1-3-5- | T | T |
| 66 | C-2 TZEQI-91 | TZE-Y Pop STR C0 S6 Inb 142 x Pool 18 SR QPM BC2S6 4-35-5-8-4-8- | T | T |
| 67 | C-3 TZEQI-74 | TZE-COMP5-Y C6S6 Inb 10 x Pool 18 SR QPM BC2S6 2-2-1-1- | T | T |
| 68 | C-4 TZEQI-82 | TZE-COMP5-Y C6S6 Inb 25 x Pool 18 SR QPM BC2S6 2-3-1-1-6-6- | T | T |
| 69 | C-5 TZEI-129 | TZE-Y Pop STR Co S6 Inbred 16-1-3- | T | T |
| 70 | C-6 TZEI-24 | TZE-Y Pop STR Co S6 Inbred 142-2-2- | T | T |

C-1, 2, 3, 4, 5 and 6 = checks 1, 2, 3, 4, 5 and 6 respectively; T= tolerance; S= susceptibility; - = data not available.
